# Manganese-enhanced MRI depicts a reduction in brain responses to nociception upon mTOR inhibition in chronic pain rats

**DOI:** 10.1186/s13041-020-00687-1

**Published:** 2020-11-23

**Authors:** Myeounghoon Cha, Songyeon Choi, Kyeongmin Kim, Bae Hwan Lee

**Affiliations:** 1 Department of Physiology, Yonsei University College of Medicine, 50-1, Yonsei-ro, Seodaemun-gu, 03722 Seoul, Republic of Korea; 2 Brain Korea 21 PLUS Project for Medical Science, Yonsei University College of Medicine, 03722 Seoul, Republic of Korea

**Keywords:** MEMRI, mTOR, Chronic pain, Torin1, XL388

## Abstract

Neuropathic pain induced by a nerve injury can lead to chronic pain. Recent studies have reported hyperactive neural activities in the nociceptive-related area of the brain as a result of chronic pain. Although cerebral activities associated with hyperalgesia and allodynia in chronic pain models are difficult to represent with functional imaging techniques, advances in manganese (Mn)-enhanced magnetic resonance imaging (MEMRI) could facilitate the visualization of the activation of pain-specific neural responses in the cerebral cortex. In order to investigate the alleviation of pain nociception by mammalian target of rapamycin (mTOR) modulation, we observed cerebrocortical excitability changes and compared regional Mn^2+^ enhancement after mTOR inhibition. At day 7 after nerve injury, drugs were applied into the intracortical area, and drug (Vehicle, Torin1, and XL388) effects were compared within groups using MEMRI. Therein, signal intensities of the insular cortex (IC), primary somatosensory cortex of the hind limb region, motor cortex 1/2, and anterior cingulate cortex regions were significantly reduced after application of mTOR inhibitors (Torin1 and XL388). Furthermore, rostral-caudal analysis of the IC indicated that the rostral region of the IC was more strongly associated with pain perception than the caudal region. Our data suggest that MEMRI can depict pain-related signal changes in the brain and that mTOR inhibition is closely correlated with pain modulation in chronic pain rats.

## Introduction

Neuropathic pain arises from an initial injury, such as neuropathy caused by a lesion of or damage to the somatosensory nervous system, that can lead to chronic pain [1]. While identifying brain abnormalities underlying chronic pain sensation could be a first step in clinical treatment, investigation of changes in cerebral neuronal activity is a challenge for functional imaging. Nevertheless, current manganese-enhanced magnetic resonance imaging (MEMRI) tools have provided a viable method for visualizing cortical responses after evoked and/or spontaneous pain [2, 3]. The chemical properties of Mn^2+^ resemble those of Ca^2+^, and Mn^2+^ acts as a paramagnetic neuronal tract tracer, as its transport uses voltage-gated Ca^2+^ channels throughout the nervous system [4–6]. Experimental hyperalgesia produces the up-regulated neuronal activation of brain responses within pain-processing regions, including the anterior cingulate cortex (ACC), insular cortex (IC), and primary (SI) and secondary somatosensory cortices (SII) [7–9]. With MEMRI studies having been found useful in identifying pain pathways in the brain, various pain imaging studies have been performed in the spinal cord and brain [5, 6], allowing researchers to visualize nerve injury [10] and thermal [11] stimulus-induced abnormal brain activities. Similarly, our previous formalin-induced pain study also showed that MEMRI could be a good indicator of pain-induced changes in the central nervous system (CNS).

Mammalian target of rapamycin (mTOR), a serine-threonine protein kinase, is known to regulate mRNA translation in the nervous system, and it plays important roles in cell proliferation and differentiation [12]. Although the roles of the mTOR pathway in cancer research have been extensively studied, there has been no in-depth study on the role of mTOR signaling in pain research. Recent studies have reported that neuropathic pain is related to structural changes in the CNS after nerve injury, known as mTOR-mediated neuronal plasticity [13–16]. The activation of mTOR regulates protein synthesis and influences a wide range of physiological and pathological states by phosphorylating downstream effectors [12, 17, 18]. In this study, the mTOR selective inhibitors, Torin1 [19, 20] and XL388 [21], were used to modulate mTOR regulator: each agent has highly potent and ATP-competitive mTOR inhibitory effects.

Here, we investigated changes in cerebrocortical excitability in nerve injury-induced chronic pain using MEMRI and compared noxious stimulation-dependent regional signal changes after application of the mTOR inhibitors using Mn^2+^ enhancement in rats. In particular, we analyzed the distributions of Mn^2+^ enhancement in the rostral and caudal parts of regions of interest (ROI) shown to be associated with different roles in pain perception. These findings may contribute to the knowledge of hyperalgesia in the brain as the result of nerve injury-induced chronic pain and may suggest potential therapeutic targets against mTOR in chronic pain.

## Materials and methods

### Animal preparation

**Fig. 1:**
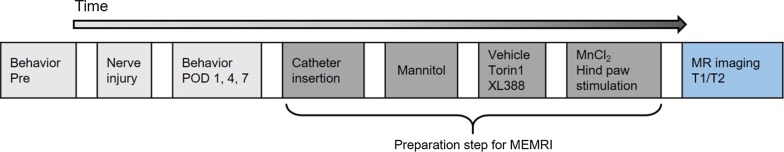
Schematic illustrating the experimental design. All rats underwent behavioral testing prior to nerve injury and at postoperative days (POD) 1, 4, and 7. On each behavioral testing day, the mechanical threshold was assessed using an electronic von Frey filament. At POD 7, rats were prepared for MEMRI experiments to determine Mn-enhanced changes after evoked pain. MEMRI was performed following T2 scans, and regional Mn enhancement in the whole brain of experimental groups was compared

Male Sprague–Dawley rats (n = 24, 250–300 g, Koatech, Pyeongtaek, Korea) were used in this study. All animal experiments were approved by the Institutional Animal Care and Use Committee (IACUC) of the Yonsei University Health System (protocol number 2016–0061). All experiments were performed in accordance with the IACUC guidelines and regulations. Animals were housed in plastic cages with soft bedding on a 12-h light/dark cycle (light cycle: 08:00 ~ 20:00), at a constant temperature (22 ± 2℃) and humidity (50 ± 10%). The experimental procedures according to the time sequence are summarized in Fig. 1. Rats were anesthetized with an intraperitoneal (i.p.) injection of sodium pentobarbital (50 mg/kg). Deep anesthesia was verified by loss of nociception in response to a tail pinch stimulation. The surgical procedure for nerve injury was performed following the methods described in our previous report [22, 23]. Briefly, the left sciatic nerve was exposed, and three major divisions were clearly separated. Then, the tibial and sural nerves were tightly ligated and transected. Complete hemostasis was confirmed, and the wound was closed with muscle and skin sutures.

Changes in mechanical paw withdrawal threshold were measured before nerve injury (pre) and at 1, 4, and 7 days after nerve injury. Rats were habituated for 10 min to the testing cages, which consisted of metal mesh floors under plastic domes. Mechanical allodynia was measured by assessing thresholds for hind paw withdrawal upon stimulation with an electrical von Frey filament (Ugo Basile, Varese, Italy). Mechanical forces were recorded with each withdrawal. Responses were measured seven times, and the means were calculated after the maximum and minimum values were excluded (Additional file 1: Fig. S1).

### Agent administration

On postoperative day 7, all rats were anesthetized with urethane (0.5 g/kg, i.p.) and α-chloralose (25 mg/kg). In addition, atropine (20 µg/kg, i.p.) was injected to avoid excessive mucus secretion in the trachea. The depth of anesthesia was determined by a lack of flexor muscle response to hind-paw pinching. Then, the rats were mounted on the surgical stage. Skin incision was performed on the right ventral aspect of the neck, and the common carotid artery and external carotid artery (ECA) were gently exposed. Polyethylene tubing (PE-10) was cannulated into the ECA.

Following catheter insertion, a 20% D-mannitol solution (35 ± 2 ℃, 5 ml/kg, Dai Han Pharm, Seoul, Korea) was infused to open the blood–brain barrier. To investigate pain-dependent signal changes in the whole brain following mTOR inhibition, nerve-injured rats were divided into three groups (Control, n = 8; Torin1, n = 8; and XL388, n = 8). Then, vehicle (0.06% DMSO in saline, 1 ml), Torin1 (400 nM in vehicle, 1 ml), or XL388 (500 nM in vehicle, 1 ml) were infused in each group of rats. Following the application, 20 mM of manganese chloride (MnCl_2_–4H_2_O, Sigma, St. Louis, MO, USA) was injected for MR imaging. All infusions were injected via the ECA (150 µl/min) using a syringe pump (22 Infusion Syringe Pump, Harvard Apparatus, Holliston, MA, USA). For noxious stimulation, electric stimulations (3 mA, 2 Hz, 1 ms width; A385, WPI, Sarasota, FL, USA) were applied to the left hind paw during manganese chloride injection.

### Magnetic resonance measurements

Magnetic resonance imaging (MRI) was performed using a 9.4-T horizontal Biospec small bore scanner (Bruker BioSpin, Ettlingen, Germany) with an 86-mm volume coil to transmit the radio frequency and a four-channel array coil to receive the signal. After tuning and shimming, T2 multi-slice spin echo sequence images were acquired for positioning. T1W images were taken at the same positions. High-resolution anatomical images were obtained with a rapid acquisition with relaxation enhancement (RARE) protocol using the following parameters: effective TE = 28 ms, TR = 4750 ms, FOV = 32 × 32 mm, matrix = 192 × 192, and RARE factor = 8. Fifteen slices of T1W images were acquired using the RARE protocol with a shortened TR. The imaging parameters were RARE factor = 2, TE/TR = 8/412 ms, FOV = 32 × 32 mm, matrix = 192 × 192, and slice thickness = 1 mm. Respiration rates of animals were monitored during the experiments. Functional analysis was performed using ParaVision (Version 5.0, Bruker BioSpin). The whole brain was outlined on axial slices, and the mean signal was calculated. To quantify data from the series of T1W images and to measure Mn^2+^ enhancement in separated brain regions, ROIs were set to entire brain regions covered in coronal images. ROIs at the insular cortex (IC), primary somatosensory cortex of the hind limb (S1HL), motor cortex 1/2 (M1/2), anterior cingulate cortex (ACC), and visual cortex 1/2 (V1/2) were manually drawn on each image. Signal magnitudes from each brain region were normalized to signal intensity in the temporalis muscle. Image analysis was performed using ParaVision (Bruker Biospin), MRVision (MRVision Co., Winchester, MA, US), and ImageJ software (National Institutes of Health, Bethesda, MD, US). Data are presented as a mean ± standard error of the mean (SEM). After MRI scans were taken, the rats were humanely killed with an overdose of urethane (1.5 mg/kg).

### Statistical analysis

To quantify the data from T1W images and the measurement of Mn^2+^ enhancement in the brain, each ROI was set to the brain atlas covered in coronal images. Relative signal intensities were calculated and compared within groups. Data are presented as a mean ± SEM. One-way analysis of variance (ANOVA) followed by Dunnett’s post-hoc pairwise comparisons were used to identify mean differences in contrast at the ROIs. All statistical analyses were performed with Prism 5 software (Version 8.03; GraphPad software Inc, San Diego, CA, US). *P* < 0.05 indicated a statistically significant difference.

## Results

### Analysis of Mn-enhanced magnetic resonance signals

**Fig. 2:**
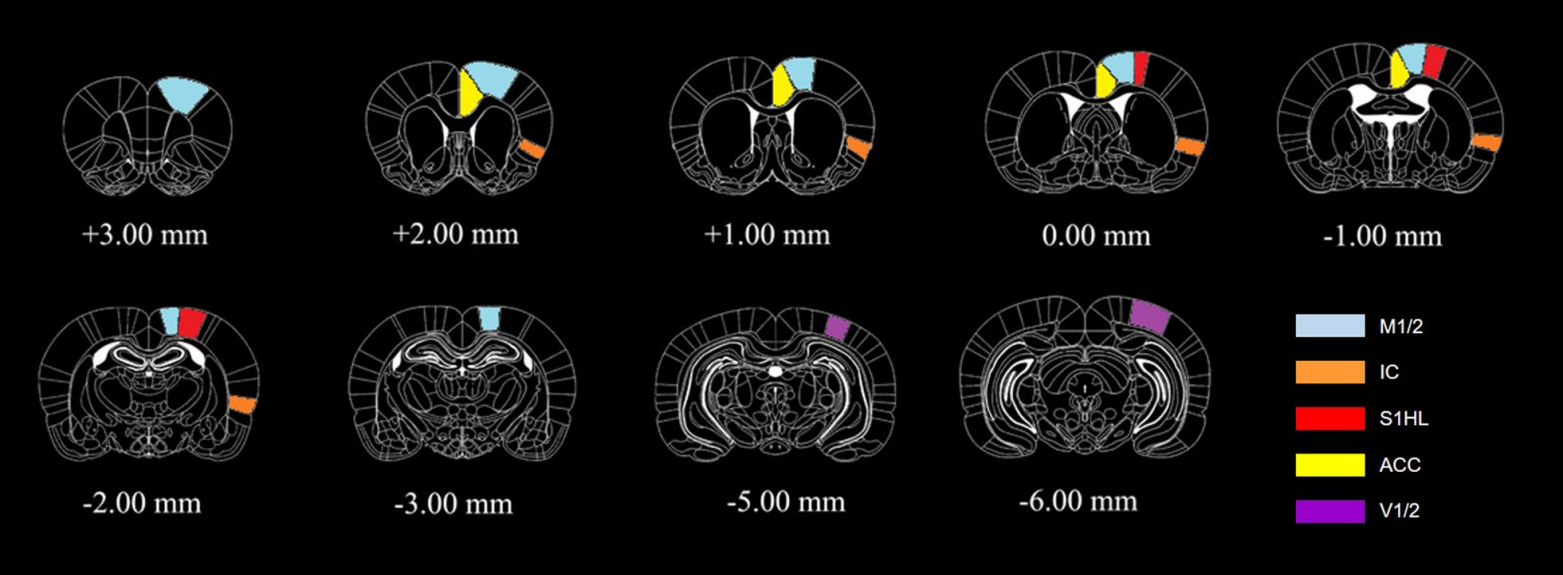
Representative images and colors of ROIs used to calculate average signal intensities. The selected ROIs are shown overlaid on T1 scans in coronal slices (anterior to posterior, respectively). M1/2: motor cortex 1/2, IC: insula, S1HL: primary somatosensory cortex hind limb region, ACC: anterior cingulate cortex, V1/2: visual cortex 1/2. Distances indicated are measurements from bregma according to the Paxinos and Watson rat brain atlas [48]

**Fig. 3:**
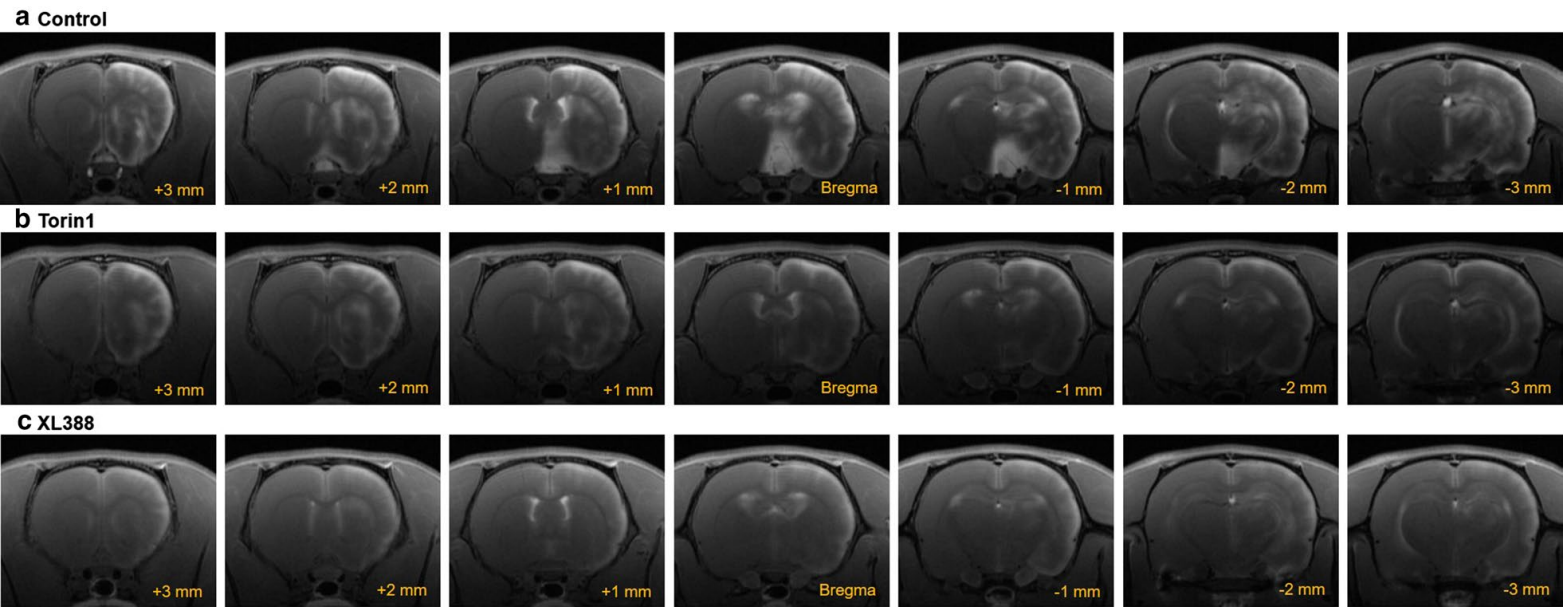
Comparison of T1-weighted MEMRI following MnCl_2_ injection in each group. MR imaging was performed for separate groups: control (vehicle), Torin1-, and XL388-infused with electrical stimulation in the hind paw. In control rats, Mn-enhanced signals were observed in the right hemisphere of the brain. However, Torin1- or XL388-infused rats did not show region-specific increases in Mn-signals in the brain. Images were obtained from bregma 3 mm to − 3 mm MRI scan

All of the acquired brain images exhibited regional heterogeneity in signal distribution typical of MnCl_2_ administration. The diffusion of Mn^2+^ was found in the medial ventral region of the brain, hypothalamus, and specific pain-related structures. High-signal intensities were observed in the IC, S1HL, M1/2, and ACC (Fig. 2). V1/2 areas were also collected as a stimuli-independent ROI. Figure 2 illustrates ROIs from which average signal intensity was extracted (several ROIs extend over several slices). In Fig. 3, the slice-dependent Mn-enhanced images of each group are compared (Fig. 3). During the comparison, high Mn-enhanced signals were observed in control (vehicle-infused) rats in all of the acquired slices. However, Torin1- and XL388-injected rats did not present pain-related Mn-enhanced signals in the IC, S1HL, M1/2, or ACC regions.

**Fig. 4:**
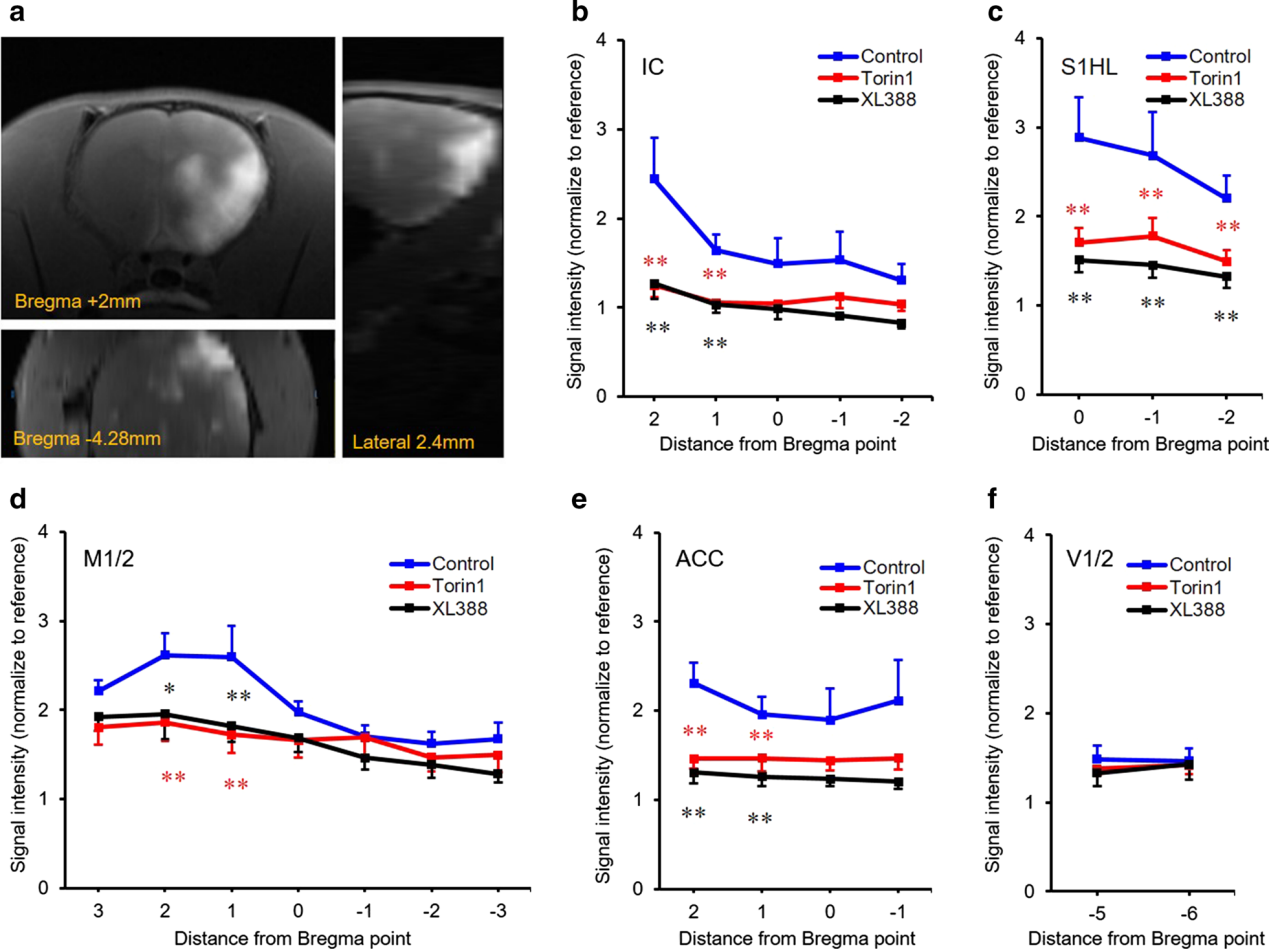
Mn-enhanced rat brain and regional signal intensity changes. **a** Following administration of MnCl_2_, there was a heterogeneous signal distribution, as shown in the coronal (left upper), horizontal (left bottom), and sagittal planes (right) of the rat brain. **b**–**f** Line graphs depict the means and standard errors for Mn-enhanced signal intensity in each brain region of the control (blue lines), Torin1 (red lines), and XL388 (black lines) rats. Five regions of Mn-enhanced signal intensity in MR images were analyzed and compared. Signal intensities in Torin1- and XL388-injected rats were lower than those in control (vehicle injected) rats. *P < 0.05; **P < 0.01. Data are presented as a mean ± SEM

Figure 4a shows the representative coronal (Fig. 4a upper left), sagittal (Fig. 4a right), and transverse (Fig. 4a left bottom) images of control rats (Fig. 4a). Across the brain regions, Mn-enhanced signals were significantly enhanced in the contralateral for nerve injury and peripheral stimulation. In the control group, the IC, S1HL, M1/2, and ACC showed higher mean signal intensities, compared to other groups. This indicated that large quantities of Mn^2+^ ions were transported and accumulated in each region. Regional signal intensity comparisons are presented in Fig. 4b–f (Fig. 4b: IC, 4C: S1HL, 4D: M1/2, 4E: ACC, and 4F: V1/2). In contrast, signal intensities in Torin1- and XL388-infused rats were lower in the IC, S1HL, M1/2, and ACC (P < 0.05 for significant differences between drugs versus control group [ANOVA followed by Dunnett’s post-hoc test]). Mean signal intensities in the IC, S1HL, M1/2, and ACC did not differ, however, between Torin1 and XL388 groups. The mean signal intensities of the V1/2 remained less-enhanced than those in other ROIs and were similar among the three groups, with no statistically significant differences (Fig. 4f). The signal intensities of the ipsilateral primary somatosensory cortex of jaws (S1J) were higher in all groups. However, it was not related to the pain caused by the given hind paw stimulus, and was excluded from data analysis. All signal intensity values are presented in Table 1.

**Table 1:** Mean signal intensities of ROIs

Region	Group	Distance from bregma								
		3 mm	2 mm	1 mm	0	− 1 mm	− 2 mm	− 3 mm	− 5 mm	− 6 mm
IC	Control		2.44 ± 0.47	1.64 ± 0.17	1.49 ± 0.29	1.54 ± 0.32	1.31 ± 0.18			
	Torin1		1.25 ± 0.13**	1.05 ± 0.12**	1.04 ± 0.08	1.12 ± 0.12	1.04 ± 0.08			
	XL388		1.27 ± 0.17**	1.03 ± 0.09**	0.98 ± 0.12	0.91 ± 0.04	0.83 ± 0.06			
S1HL	Control				2.89 ± 0.45	2.68 ± 0.49	2.20 ± 0.25			
	Torin1				1.71 ± 0.16**	1.78 ± 0.21**	1.49 ± 0.13**			
	XL388				1.51 ± 0.14**	1.45 ± 0.14**	1.32 ± 0.12**			
M1/2	Control	2.53 ± 10.37	2.85 ± 9.35	2.85 ± 0.41	2.34 ± 0.36	2.08 ± 0.39	1.68 ± 0.13	1.72 ± 0.18		
	Torin1	1.81 ± 0.19	1.86 ± 0.21**	1.72 ± 0.21**	1.66 ± 0.19	1.69 ± 0.23	1.47 ± 0.16	1.50 ± 0.20		
	XL388	1.92 ± 0.16	1.95 ± 0.28*	1.82 ± 0.18**	1.69 ± 0.16	1.46 ± 0.13	1.39 ± 0.15	1.28 ± 0.10		
ACC	Control		2.31 ± 0.23	1.96 ± 0.19	1.89 ± 0.36	2.11 ± 0.46				
	Torin1		1.93 ± 0.48**	1.93 ± 0.48**	1.91 ± 0.48	1.92 ± 0.47				
	XL388		1.30 ± 0.12**	1.26 ± 0.10**	1.23 ± 0.08	1.20 ± 0.08				
V1/2	Control								1.49 ± 0.15	1.46 ± 0.15
	Torin1								1.38 ± 0.07	1.42 ± 0.10
	XL388								1.33 ± 0.14	1.43 ± 0.17

Comparison of Control vs. Torin1 or XL388 (*P < 0.05 and **P < 0.01)

### Comparison of Mn-enhanced signal expression

**Fig. 5:**
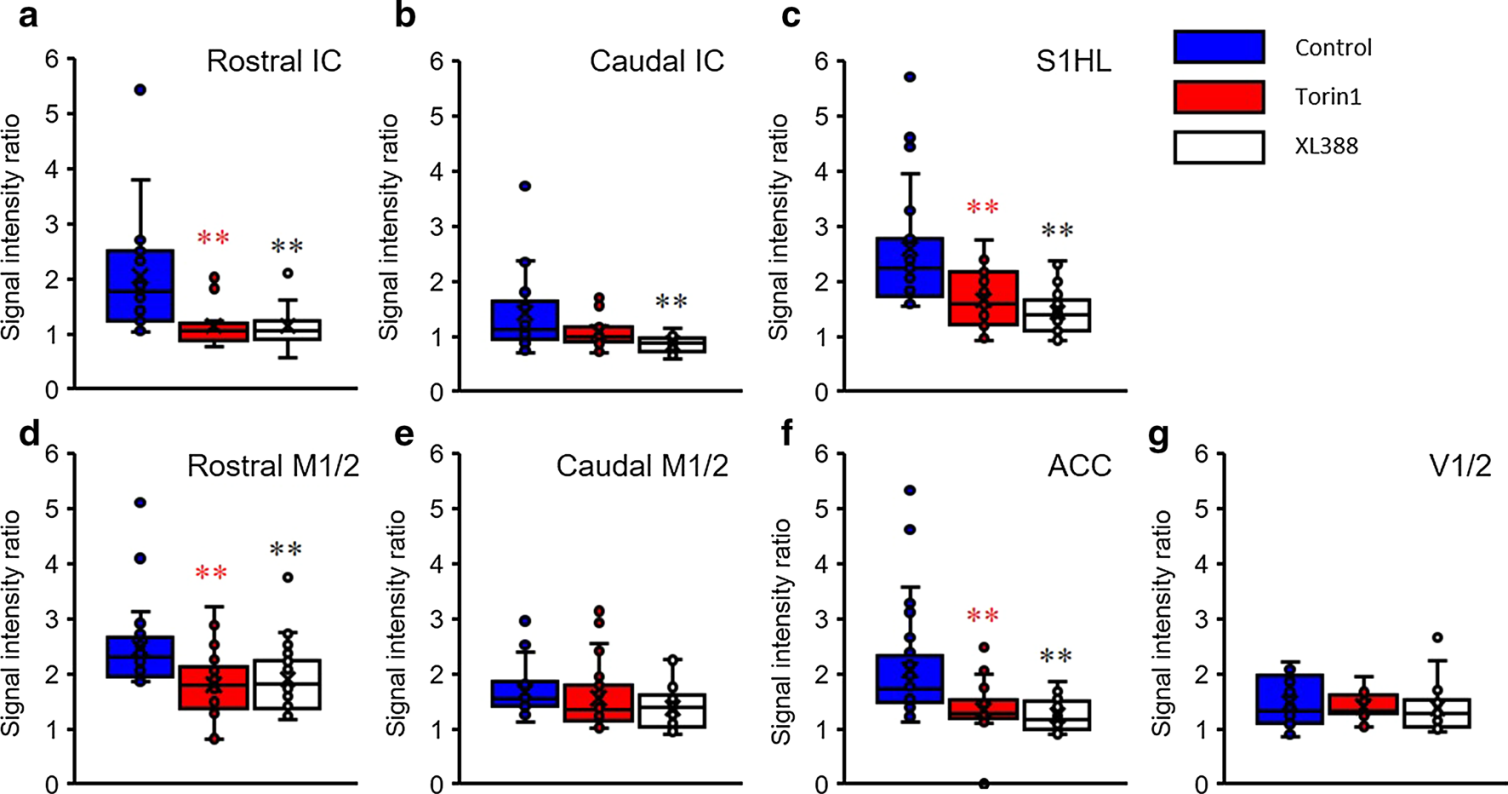
Comparison of Mn^2+^ enhancement in the ROIs after noxious stimulation. The IC regions were analyzed by rostral-caudal analysis (**a**, **b**). S1HL regions showed increased signals in all groups. However, Torin1-, or XL388-treated rats showed reduced signal intensities (**c**). Torin1- and XL388-treated rats showed significantly reduced signal intensities at the rostral M1/2 area (**d**, **e**). In the comparison of ACC, Torin1- and XL388-treated groups showed a significant reduction in signal enhancement (**f**). However, V1/2 signals did not show any difference within groups (**g**). Each group is presented by different colors (control: blue, Torin1: red, and XL388: white). *P < 0.05; **P < 0.01. Data are presented as a mean ± SEM

To elucidate the differences between groups, we analyzed the integrated signal values of ROIs, and the IC and M1/2 regions were divided into rostral-caudal parts and analyzed. The comparison of regional signal intensities between groups is presented in Fig. 5. Stimulus-dependent Mn-enhanced signal intensities were significantly reduced after Torin1 and XL388 infusion, and Mn-enhanced signals after hind paw stimulation in the IC appeared higher in the rostral planes (Fig. 5a, bregma 2 and 1 mm) than in the caudal planes (Fig. 5b, bregma − 1 and − 2 mm) of the IC. In the S1HL, which directly received painful nociception signals after stimuli, we observed increased signal values in the whole plane in the control group. However, significant reductions were observed in the Torin1- or XL388-infused rats (Fig. 5c). In the M1/2 rostral-caudal comparison, Torin1- and XL388-infused rats showed regional specific Mn-enhancement in the M1/2 area, compared to control rats (Fig. 5d and e). At the caudal part of the M1/2 (Fig. 5e, bregma − 1, − 2, and − 3 mm), there was no significant difference between groups. However, for the rostral part of M1/2 (Fig. 5d, bregma 2 and 1 mm), data from Torin1- or XL388-injected groups showed significantly lower values (Fig. 5d, ANOVA followed by Dunnett’s post-hoc test). In addition, in the comparison of signal intensities in the ACC, Torin1- or XL388-treated groups showed significantly reduced signal enhancement in the rostral part (Fig. 4f, bregma 2 and 1 mm). However, the V1/2 signal intensities in pain-independent brain regions showed no difference in any group.

### Stimulus-induced signal intensities in ROIs

**Fig. 6:**
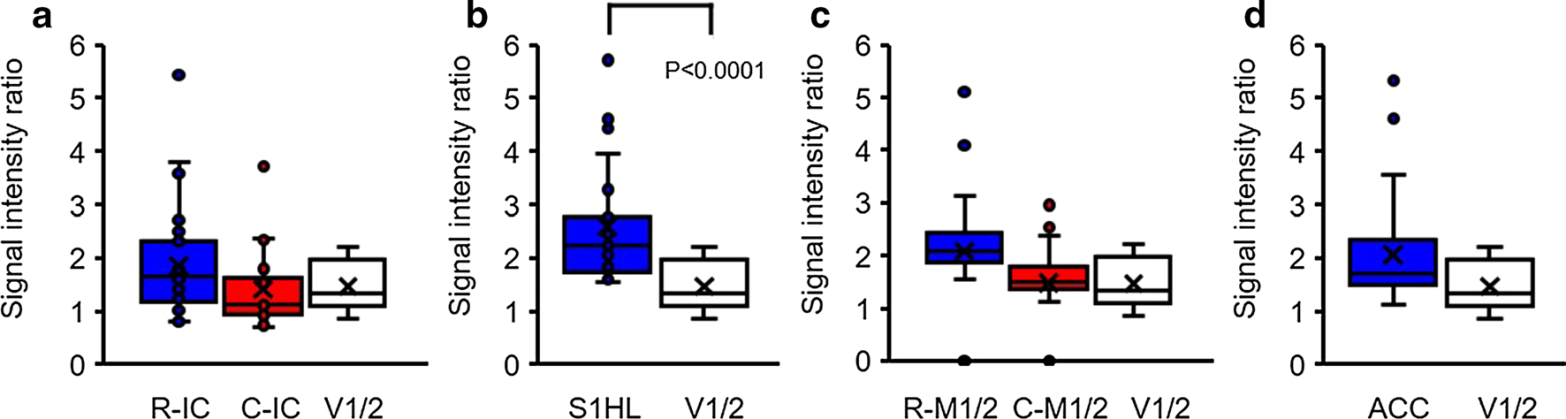
Analysis of stimulation-dependent ROIs in control rats. Relative manganese-enhanced MRI-T1-contrast for the investigated insular (rostral and caudal), S1HL, M1/2 (rostral and caudal), and ACC regions of the brain in control (blue or red columns), compared to visual cortex (white columns). Each *P* value indicates a significant difference between groups. Data are presented as a mean ± SEM

In order to quantify the stimulus-dependent signal intensity changes, signal intensities at ROIs were compared to the signal intensities of the V1/2 regions of the same animals (Fig. 6). In signal intensity comparisons between the rostral area of the IC and the caudal area of the IC, the rostral area of the IC showed more enhanced signal activities when painful stimuli were given to the hind paw, compared to the caudal area of the IC. This signal enhancement was more explicit when compared to the V1/2 (Fig. 6a; rostral-IC 2.04 ± 0.28, caudal-IC 1.42 ± 0.19, and V1/2 1.47 ± 0.11). In addition, the S1HL directly reflected hind paw stimulation, and signal differences with V1/2 were greater than those in other regions (Fig. 6b; S1HL 2.59 ± 0.25, and V1/2 1.47 ± 0.11). In comparison of mean signal enhancement in the M1/2 region, the rostral part of the M1/2 region showed more enhanced activities, compared to the caudal part of M1/2 region (Rostral-M1/2 2.20 ± 0.22 vs. Caudal-M1/2 1.48 ± 0.14). However, the difference was not significant in comparison with the V1/2 region. In addition, the ACC also showed no significant difference compared to the V1/2 signal intensities (Fig. 6d ACC 2.07 ± 0.17).

## Discussion

The purpose of this study was to evaluate the usefulness of MEMRI in chronic pain research using whole brain analysis, as well as to measure changes in brain activities after drug administration in a chronic pain model. In this study, we demonstrated cortico-cortical neural excitation of several ROIs under chronic neuropathy using MEMRI, as well as changes in Mn-enhanced signals of the pain-related cortex area after direct infusion of mTOR inhibitors in the brain. Until now, the effects of mTOR inhibitor using animal MRI have not been studied. Several studies have used the MEMRI method to assess pain in the central nervous system or to observe changes in pain through analgesic use [3, 10, 24]. However, the MEMRI method has several difficulties in observing the effects of pain reduction or changes in neuronal activities. In order to abbreviate these problems, we tried to observe dynamic changes in the brain using ECA injection with MnCl_2_.

### *The meaning of Mn*^*2*+^*enhancement in ROIs*

MEMRI has advantages of a fast influx and enhancements of Mn^2+^ in the tissues [24]. In addition, the cumulative Mn^2+^ pattern is retained for several days, and it also provides a good opportunity to observe activated brain changes upon stimulation when viewed through T1-weighted MRI [5]. Quantitative evaluation of contrast enhancement showed that MnCl_2_ injection via ECA may be the best way to show hind paw stimulation-induced pain in rats, with the best signal-to-noise ratio [2]. The Mn-injection method through ECA could observe the signal changes only on the ipsilateral side, which is related to blood circulation in the brain [25]. In order to observe whole brain activation with MEMRI, Mn-injection through subarachnoid or direct injection into the brain regions were also considered. Nevertheless, Mn-injection through the ECA for MEMRI was considered to better facilitate the observation of specific structures and activity, rather than mere signal enhancement by Mn-diffusion.

Reliable brain activities were observed using the MEMRI method in the control, Torin1-, and XL388-infused groups. Mn^2+^ enhancement of individual slices was measured and analyzed in the rostral and caudal regions. Integrated signal analysis of the IC showed higher activated signals in the rostral region than in the caudal region of control animals. These results were consistent with previous findings that the rostral part of the IC is more involved in pain processing [26, 27]. A previous anatomical study [26] revealed that the rostral part of the IC receives considerable input from the centrolateral and mediodorsal thalamus, which showed strong activity in response to noxious stimulus in MEMRI results. The functional characteristics of the caudal part of IC were clearly distinct from those of the rostral part of IC, which has received attention for its role in nociceptive processing [28]. The rostral part of IC directly accepts the pain caused by stimulation, while the caudal part of IC may function pathologically with persistent maintenance of mechanical allodynia [29]. In this study, rats infused with Torin1 or XL388 showed not only a reduction of signals in the rostral part of IC, but also a reduction of signals in the caudal part of IC. In addition to the IC, other cortical target regions, such as the S1HL and ACC, have been investigated to evaluate the pain inhibition effects after medication. In the S1HL, which directly reflects noxious stimulation, subcutaneous electrical stimulation in the hind paw could activate Aβ fibers from cutaneous receptors [30]. Evoked pain sensation was reflected in the S1HL. In addition, the ACC was consistently activated in nociception in human and animal studies [31]. Previous research has indicated that the neuronal plasticity in ACC is highly correlated with the development of chronic pain [32, 33]. Although the ACC has been shown to be associated with different aspects of pain, precise functional mapping of these aspects still remains largely unknown.

Interestingly, we observed enhanced signals in the M1/2 region upon noxious hind paw stimulation. Although M1/2 is not a part of the pain matrix, it is known that the M1/2 has wide connections to some of the sensory relay nuclei in the thalamus, as well as to efferent and afferent fibers in the spinal cord, that are responsible for the transmission of painful stimuli and modulation of the motor response to noxious contact. Previous investigators have indicated that the motor cortex in rats and mice partially overlaps the S1 to form a “sensorimotor amalgam” that largely involves representations of the hind limb [34–36]. Despite this knowledge, it is difficult to explain how noxious stimuli in the hind paw activate the M1/2. Further research is required to explain the role of the M1/2 in pain perception.

### How can mTOR inhibition reduce brain activities in response to chronic pain?

Modulation of chronic pain through mTOR inhibition is a newly studied target for chronic pain control [16, 37]. Previous studies have observed changes in chronic pain through the inhibition of mTOR signaling in the spinal cord [38–41], and recent studies have demonstrated effective modulation of chronic pain by direct mTOR signaling control in the brain [16, 32, 42], The activation of mTOR regulates protein synthesis by phosphorylating downstream effectors, which influence a wide range of physiological and pathological states, including neuropathic, inflammatory, and cancer-related pain [16–18]. The mTOR inhibitors Torin1 and XL388 used in this study are ATP-competitive inhibitors that suppress the phosphorylation of downstream effectors to regulate mRNA translation and protein synthesis [43]. Our previous research revealed how Torin1/XL388 can change chronic pain after nerve injury [44, 45]. In these studies, Torin1 or XL388 was microinjected into the brain of nerve-injured animals and behavioral changes were assessed. The results indicated that the administration of Torin1 or XL388 significantly increased mechanical thresholds and reduced mechanical allodynia. These results strongly suggest that Torin1 and XL388 may attenuate neuropathic pain via inhibition of mTOR in the brain. Remarkably, the Torin1- and XL388-infused rats showed reduced Mn-enhanced signals in the cortical area, including the IC, S1HL, M1/2, and ACC. These results demonstrate that mTOR inhibition may have an effect on activation of other brain regions, as well as pain reduction through the inhibition of phosphorylation by mTOR control [13, 32, 38]. The maintenance of long-term potentiation (LTP) is dependent on protein synthesis, and mTOR signaling is involved in the local protein synthesis required for the maintenance of LTP. In the process of pain-related neural plasticity, mTOR kinase is one of the key factors related to protein synthesis. Also, increases in mTOR following nerve injury has been shown to increase phosphorylation, indicating that the protein synthesis involved in neural plasticity causing neuropathic pain is increased. Our another previous research has reported that the expressions of mTOR-related translation factors, such as 4E-BP1 and p70S6K, are increased under chronic pain conditions [16]. In addition, several studies have confirmed activation of PKCα, which follows mTOR phosphorylation [46, 47]. PKCα is known to play a critical role in cytoskeleton rearrangement induced by mTOR. Moreover, down-regulation of the PKCα generates an abnormal cell shape or excessive actin cytoskeleton and alters neurons by rearranging their configuration, volume, or length. Thus, we assume that the reduced signal intensities observed in the Torin1- and XL388-treatment groups may be attributable to the relief of neuropathic pain due to inhibition of protein synthesis and cytoskeleton rearrangement associated with mTOR.

In our results, there were no differences in signal enhancement ratios between experimental groups in the V1/2 region. These results indicate that the inhibitory effect of mTOR signaling is not significantly different in response to similar visual cues. Recent studies have suggested that mTOR signaling may be involved in the initiation of pain perception [15, 19], which could effectively reduce chronic pain through inhibition of mTOR. However, the amount of research on how pain is controlled due to regulation of mTOR is still insufficient. A better understanding of these signaling pathways would provide great insights into the underlying mechanisms and lead to more refined therapeutic approaches.

In conclusion, the activity-dependent Mn-enhanced magnetic resonance signals after mTOR inhibition reported herein indicated that pharmaceutical inhibition of mTOR signaling in the brain could reduce abnormal cortex activities after chronic neuropathic pain. Furthermore, rostral-caudal analysis revealed that the rostral regions of IC and M1/2 are more related to pain perceptions than caudal regions. Nevertheless, MRI studies with manganese ions have many challenges, due to the restrictive pass capability of the blood–brain barrier, in addition to difficulties in controlling the accumulation of manganese in tissues and physiological changes in the body. The development of various studies using improved MR techniques, real-time optical imaging with voltage-sensitive dye, and brain slice recordings may help contribute to future studies of neural mechanisms and help to advance knowledge of cerebral activation in chronic pain.

## Supplementary information

**Additional file 1: Fig. S1.** Pain behaviors assessed before injury and at POD 1, 4, and 7. There was no change in hind paw withdrawal thresholds following sham nerve injury either before injury or at POD 1, 4, and 7. However, for nerve-injured rats, there was a significant reduction in withdrawal thresholds after nerve injury. Data were analyzed using a paired t-test and two-tailed post-hoc test; error bars represent the standard error of the mean; *P < 0.05, ***P < 0.0001.

## Data Availability

The datasets used and/or analyzed during the current study are available from the corresponding author on reasonable request.
