# Supplementary figures and images for: Manganese-enhanced MRI depicts a reduction in brain responses to nociception upon mTOR inhibition in chronic pain rats
Source: Mol Brain. 2020 Nov 23;13:158. doi: 10.1186/s13041-020-00687-1 (PMC7713325; doi:10.1186/s13041-020-00687-1)

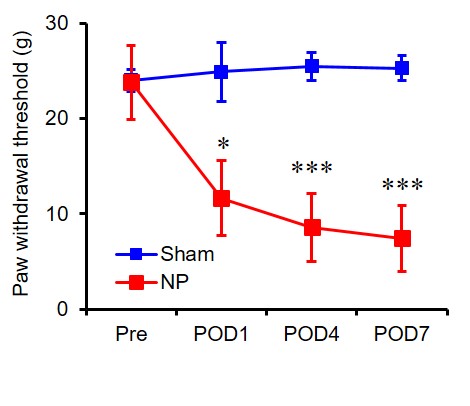

Supplement: Supplementary file 1 — Additional file 1: Fig. S1. Pain behaviors assessed before injury and at POD 1, 4, and 7. There was no change in hind paw withdrawal thresholds following sham nerve injury either before injury or at POD 1, 4, and 7. However, for nerve-injured rats, there was a significant reduction in withdrawal thresholds after nerve injury. Data were analyzed using a paired t-test and two-tailed post-hoc test; error bars represent the standard error of the mean; *P < 0.05, ***P < 0.0001. [file 13041_2020_687_MOESM1_ESM.jpg]
